# We have the program, what now? Development of an implementation plan to bridge the research-practice gap prevalent in exercise oncology

**DOI:** 10.1186/s12966-020-01032-4

**Published:** 2020-10-09

**Authors:** Mary A. Kennedy, Sara Bayes, Robert U. Newton, Yvonne Zissiadis, Nigel A. Spry, Dennis R. Taaffe, Nicolas H. Hart, Michael Davis, Aileen Eiszele, Daniel A. Galvão

**Affiliations:** 1 Exercise Medicine Research Institute, Edith Cowan University, 270 Joondalup Drive, JOONDALUP, Perth, WA 6027 Australia; 2 School of Medical and Health Sciences, Edith Cowan University, Perth, WA Australia; 3 School of Nursing, Midwifery and Paramedicine, Australian Catholic University, Fitzroy, VIC Australia; 4 School of Nursing and Midwifery, Edith Cowan University, Perth, WA Australia; 5 School of Human Movement and Nutrition Sciences, University of Queensland, Brisbane, QLD Australia; 6 GenesisCare, Perth, WA Australia; 7 Faculty of Medicine, University of Western Australia, Perth, WA Australia; 8 Institute for Health Research, University of Notre Dame Australia, Fremantle, WA Australia; 9 Cancer and Palliative Care Outcomes Centre, Queensland University of Technology, Brisbane, QLD Australia

**Keywords:** Cancer, Physical activity, Knowledge translation, Organizational change, Chemotherapy, Radiotherapy

## Abstract

**Background:**

Exercise has emerged as a promising therapy for people with cancer. Novel programs have been developed to translate research into practice; however, implementation barriers have limited their success in part because successful translation of exercise oncology research into practice requires context-specific implementation plans. The aim of this study was to employ the implementation mapping protocol to develop an implementation plan to support programming of a co-located exercise clinic and cancer treatment center.

**Methods:**

The Implementation Mapping protocol, which consists of five specific iterative tasks, was used. A stakeholder advisory group advised throughout the process.

**Results:**

A comprehensive needs assessment was used to identify the organization’s general manager as the program adopter; oncologists, center leaders, and various administrative staff as program implementers; and the operations manager as the program maintainer. Twenty performance objectives were identified. The theoretical domains framework was used to identify likely determinants of change, which informed the selection of eight individual implementation strategies across the individual and organizational levels. Finally, an evaluation plan was developed which will be used to measure the success of the implementation plan in the project’s next phase.

**Conclusion:**

The Implementation Mapping protocol provided a roadmap to guide development of a comprehensive implementation plan that considered all ecological domains, was informed by theory, and demonstrated an extensive understanding of the implementation context. Strong research-practitioner partnerships and effective stakeholder engagement were critical to development of the plan.

## Introduction

Evidence supporting the benefits of exercise for people with cancer has grown exponentially over the past two decades, demonstrating a clear benefit (e.g., decreased fatigue, increased health-related quality of life) for several common side-effects of treatment suggesting a potential role in enhancing the benefits of treatment [1–3] and leading to an updated version of exercise guidelines for cancer survivors from national and international organisations in 2019 [4, 5]. This compelling evidence base has led to the recommendation of exercise being considered a standard component of care in oncology. However, few patients report engaging in meaningful amounts of exercise, with estimates ranging between 18 and 47% meeting general guidelines [6, 7].

Given the potential therapeutic impact of exercise, calls have been made to make exercise part of routine care for patients [8]. Incorporating exercise into healthcare has the potential to increase patient participation by relying on a trusted physician to introduce the importance of exercise at a time when patients are willing to engage in new health behaviors (i.e., a teachable moment) [9]. Yet, despite strong patient desire to receive exercise guidance from an oncology care team member [10] and widespread clinician support for the need to recommend exercise to patients [11], clinician-driven exercise referrals rarely occur in practice [12]. The result is that exercise oncology is caught in the research-to-practice gap, where research has demonstrated the therapeutic potential of exercise, but has not developed systems to connect patients to effective programs. Novel approaches are needed to bridge the gap.

One example of a program designed to bridge the practice gap is the co-located exercise clinic (Co-LEC) at GenesisCare Western Australia. GenesisCare, a private oncology clinic, partnered with the Exercise Medicine Research Institute (EMRI) at Edith Cowan University (ECU) to co-locate an exercise clinic within their cancer treatment facility. The concept was to facilitate oncologist referrals to exercise by providing a clear pathway to a trusted exercise professional. However, an evaluation of the service revealed it was underutilized despite providing effective programming for patients and being a good organizational fit for GenesisCare [13]. Its poor utilization appeared to be largely due to the lack of an implementation plan. No work was undertaken to design an implementation protocol to fit a new context; instead, the team relied on systems designed for the EMRI research and exercise clinic. In the end, the systems were not functional in a dynamic private healthcare setting.

Implementation science has demonstrated that, to be successful, evidence-based programs need to be supported by evidence-based implementation strategies, tailored to the specific needs of the environment in which they will be employed (i.e., context) [14]. Recognizing the Co-LEC’s effectiveness and organizational fit, the aim of this study was to describe an implementation plan specific to the GenesisCare context to overcome the utilization issues. The Implementation Mapping (IM) protocol was used to guide the project [15]. IM was chosen because this systematic process takes an ecological approach and guides implementation design using stakeholder input, ensuring specificity to the context in which it will be employed [16]. Understanding how to effectively implement the Co-LEC improves access to exercise services for GenesisCare patients. More broadly, this work provides insight into how to create context-specific implementation plans to facilitate integration of effective exercise programs into the routine care of cancer patients. This work helps bridge the research-to-practice gap currently limiting the expansion of exercise oncology in clinical care.

## Methods

An IM approach was used to develop an implementation plan for the Co-LEC. IM was developed by combining insights from implementation science with strategies from step 5 of the comprehensive IM protocol, which takes an ecological approach to solving problems, guiding users to make key decisions based on a combination of theory, evidence, and stakeholder input [15, 17]. IM is relevant for already developed evidence-based practices, but need a plan to be adopted, implemented, and maintained in a real-world setting [15]. While the process has been applied as a component of overall program development in exercise oncology [18], the implementation tasks have not been comprehensively described; however, the protocol has shown promise in other disciplines [19]. There are five specific tasks involved in this iterative process and each is guided by input from a stakeholder advisory group (SAG) (Table 1).

**Table 1:** Components of Implementation Mapping process

Intervention Mapping Step	Purpose	Questions addressed
**Task 1**	Engage stakeholders integral to the context where the intervention will be employed who will be responsible for its adoption, implementation, and maintenance. Identify actions necessary for and barriers/facilitators of implementation.	1. Who will decide to adopt and use the program?
Conduct a needs and assets assessment		
		2. Which stakeholders will decision makers need to consult?
		3. Who will make resources available to implement the program?
		4. Will the program require different people to implement different components?
		5. Who will ensure the program will continue as long as it is needed?
**Task 2**	Define who needs to do what to adopt, deliver, and maintain the program and its essential elements and create change objectives to guide implementation design.	4. What is are the target outcomes the adopters, implementers, and maintainers need to achieve?
Identify adoption and implementation outcomes, performance objectives, determinants, and change objectives		
		5. What needs to be done to adopt, implement, and maintain the program (i.e. performance objectives)?
		6. What personal determinants will influence the “why” decisions for each group?
		7. What has to change for each determinant in order to encourage the performance objectives?
**Task 3**	Using theory, choose implementation strategies to create practical applications for the specified context.	8. What theory-based change methods are most appropriate for this context?
Select theoretical methods and design implementation strategies		
		9. How can these change methods be used as practical applications in this context?
**Task 4**	Outline implementation plan.	10. What program components need to be created?
Produce implementation protocols and materials		
		11. Are the program materials appropriate for the target audience?
**Task 5**	Design evaluation plan.	12. What needs to be measured to describe program effects before and after the implementation?
Produce implementation protocols and materials		
		13. How will the chosen constructs be measured?

In task 1, a needs and assets assessment was conducted to determine barriers and facilitators for implementation, and to identify who is responsible for adopting, implementing, and maintaining the program within the organization. The needs assessment was informed by the RE-AIM model (Reach, Effectiveness, Adoption, Implementation, Maintenance) to ensure issues relevant to implementation were taken into account [20]. In task 2, the expected outcomes for adoption, implementation, and maintenance were defined and supported through the development of a change matrix, linking the behaviors necessary to achieve the outcomes with their determinants. In task 3, theory-informed evidence-based implementation strategies were selected to address the program objectives defined in task 2, and then practical implementation strategies were chosen to operationalize the methods. In task 4, an overall implementation plan was created with supporting materials and structures in partnership with key stakeholders. Relevant GenesisCare clinic staff were consulted throughout the design process. Finally, in task 5 an evaluation plan was designed to be used in the project’s next phase to determine the effectiveness of the implementation plan.

Ethics approval was provided by ECU’s Human Research Ethics Committee (ID: 20888 KENNEDY). Consent was obtained for all stakeholders who participated in the needs assessment. Interviewees provided written informed consent prior to their interview and survey respondents provided consent before beginning the questionnaire.

### Stakeholder advisory group

A SAG was assembled by the EMRI-ECU research and GenesisCare leadership teams. Each group identified individuals from their organization familiar with the Co-LEC to serve on the SAG. Additionally, the Consumer and Community Health Research Network (CCHRN), a non-profit organization connecting consumers with researchers, was engaged to identify people living with and beyond cancer suitable to participate in the SAG (https://www.involvingpeopleinresearch.org.au/). The group met monthly during the planning phase of the project (5 months). The SAG was comprised of 10 people: 4 EMRI-ECU exercise oncology researchers responsible for initial development and familiar with the ongoing operations of the Co-LEC, 1 EMRI-ECU implementation researcher, 3 GenesisCare leadership team members involved in the initial adoption and ongoing operations of the Co-LEC, and 2 cancer patient representatives. All data were deidentified and summarized before being shared with the SAG.

### Theoretical underpinnings

The project was nested in Grol and Wensing’s ecological framework for examining barriers and incentives for change within healthcare [21]. This framework recognizes six levels within a healthcare setting needing to be considered when planning complex changes in practice: nature of the innovation, characteristics of patients and professionals, and the social, organizational, and economic/political contexts. Professional, social, and organizational characteristics were targeted in this project, guided by the Constructs of the Theoretical Domains Framework (TDF) [22]. TDF was chosen as its original intent was to identify influences on health professional behaviors related to implementation of evidence-based changes, and because it has been successfully applied in a variety of healthcare settings to guide implementation of evidence-based interventions and guidelines [23]. The comprehensive perspective of TDF, which synthesises 33 theories of behavior and behavior change, provided a broad lens to view the potential influencers needing to be considered for this implementation project. The key domains considered were knowledge, environmental context and resources, social influences, and beliefs about consequences.

## Results

### IM task 1: needs and assets assessment

A comprehensive needs assessment of the existing Co-LEC service was developed using the RE-AIM framework. A full description of the assessment and its results are described in detail [13]. Briefly, four key stakeholder groups were identified as critical to understanding barriers and facilitators related to utilisation: GenesisCare cancer patients (including both Co-LEC participants and non-participants), GenesisCare oncologists, GenesisCare nurses and EMRI-ECU Accredited Exercise Physiologists (AEPs). Clinic records were also collected to provide perspective on overall utilization and financing of the Co-LEC service. One-hundred nineteen GenesisCare cancer patients completed a survey describing their experience with the Co-LEC, questionnaires and workout summary sheets were completed by 237 Co-LEC participants, and semi-structured interviews were conducted with 7 GenesisCare oncologists, 8 GenesisCare nurses, and 3 AEPs. The needs assessment suggested the Co-LEC concept offered a good organizational fit but had several behavioral and environmental barriers to overcome. Notably, patients who attended the service reported high levels of satisfaction but expressed frustration with logistics (e.g. inadequate hours of operation). Those who did not attend expressed a strong interest, but the majority (45%) reported not knowing it was available whilst they were undergoing treatment. Oncologists reported wanting to refer patients to the service, but not feeling confident with the referral process or satisfied with the availability of the program. Two oncologists were responsible for most of the referrals. Additionally, inefficient systems created a referral process that discouraged use, which was made worse by poor communication between the exercise and clinical oncology staff. Finally, a lack of funding resulted in service cutbacks making the service inaccessible for many patients.

The assessment clarified who needed to be targeted for the various stages of implementation. While the Co-LEC had already been adopted by GenesisCare, the general manager (GM) was defined as the person responsible to adopt the proposed implementation changes and to make resources available for the program. Multiple people were identified as responsible for implementing different components of the program: oncologists, patient services officers (PSO), AEPs, billing officers, and center leaders. The operations manager was deemed responsible for ensuring the program was maintained for as long as needed.

### IM task 2: performance and change objectives

The target adoption, implementation, and maintenance outcomes for each group (adopters, implementers, and maintainers) were defined and the specific steps required to meet them (i.e., performance objectives) were outlined (Table 2). Twenty performance objectives were identified across all groups. This work was guided by results of the RE-AIM assessment [13], which helped the SAG identify who needed to be involved in the program’s implementation plan and what actions were necessary to enhance implementation effectiveness. For example, the evaluation elucidated the critical role of oncologist referrals, the absence of effective communication strategies, and the need for a billing structure. The GenesisCare GM, a member of the SAG, identified people within the organization who would be best suited to work through those issues. Those people were contacted to contribute to the development of performance objectives. Next, the SAG consulted the literature to understand the changeable determinants within healthcare implementation that could help explain why the adopters, implementers, and maintainers would perform a particular behavior. The salient constructs of the TDF framework identified by the SAG were knowledge (program goals and procedures), group norms, environmental facilitators, and outcome expectations [22, 24, 25]. These were linked to each performance objective, creating a “change matrix” that defined what needed to change in order to achieve the performance objective and served as a blueprint for the selection of implementation strategies. Table 3 represents a portion of the change matrix developed for program implementers.

**Table 2:** Target adoption, implementation, and maintenance outcomes and performance objectives by role

Target: Role	Adoption, Implementation, and Maintenance Outcomes	Performance Objectives
**General manager (GM):**	The GM decides to adopt the Co-LEC implementation program as indicated by completing a memorandum of understanding (MOU).	1. Agree to re-implement the Co-LEC
Adopter		2. Agree to expand exercise services
		3. Approve updates to systems (e.g. electronic medical records, EMR), internal workflows, and policies necessary to support the exercise service
		4. Approve allocation of appropriate staff to support the initiative
**Oncologist:**	The oncologist will tell patients about the Co-LEC and complete a referral for all eligible patients.	1. Discuss Co-LEC service with new patients
Implementer		2. Tell the patient about the chronic disease management plan payment option
		3. Tick box to refer eligible patients to service
		4. Check-in with patients during ongoing appointments to ask about exercise progress.
**Accredited exercise physiologist (AEP):**	The AEP will integrate the service utilizing standard operating protocols for other clinicians at GenesisCare.	1. Record all Co-LEC information into the electronic medical record system
		2. Request ongoing appointments using electronic quick orders
Implementer		
**Patient services officers (PSO):**	The PSO will include Co-LEC information in all new patient packets, call eligible patients to book an initial appointment at the Co-LEC, and schedule all ongoing appointments as directed by the AEP.	1. Add the Co-LEC brochure to all new patient packets
		2. Call to schedule an initial appointment at the Co-LEC for all oncologist referrals
Implementer		
		3. Book in ongoing Co-LEC appointments based on all AEP quick orders
**Billing officer:**	The billing officer will match all CDMPs against patient appointments at the Co-LEC and bill accordingly.	1. Update billing protocol to include exercise claims
Implementer		2. Train staff regarding new procedures
**Center leader:**	The center leader will ensure all resources are available for the oncologists and PSOs.	1. Institute systems changes to EMRs and work with technology staff to make changes
Implementer		
		2. Ensure Co-LEC is properly resourced to perform optimally
**Operations manager:**	The operations manager will ensure the general manager maintains the Co-LEC as part of standard practice.	1. Monitor implementation barriers
Maintainer		2. Report key program metrics and needs to GM
		3. Advocate for program changes required to sustain program

*GM* General manager; *Co-LEC* Co-located exercise clinic; *EMR* Electronic medical records; *PSO* Patient services officers, *AEP* Accredited exercise physiologist

**Table 3:** Partial matrices of change objectives for co-located exercise clinic (Co-LEC) implementers

Behavioral outcome: Oncologist tells patients about the Co-LEC and completes a referral for all eligible patients				
**Performance objectives**	**Determinants**			
	**Knowledge**	**Group Norms**	**Environmental Facilitators**	**Outcome Expectancies**
PO.1. Oncologist discusses service with new patients	Understand the service and how it can benefit patients	Believe that other oncologists are discussing the service with their patients; it is an expectation of practice	Materials are available to remind oncologist to discuss Co-LEC service and provide talking points for discussion.	Expectation that a discussion with patient will result in patient attendance at the Co-LEC, which will positively impact their treatment experience.
PO.2. Oncologist tells patients about Medicare payment option	Be aware that Medicare is an option for payment	Believe that other oncologists are discussing Medicare payment options with their patients; it is an expectation of practice.	Materials are available to remind oncologist to discuss Medicare payment and provide talking points for discussion.	Expectation that a discussion with patient will result in utilization of Medicare payment plan.
PO.3. Oncologist ticks boxes to refer eligible patients to service	Describe the role of exercise during cancer treatment. Identify potential safety concerns for each patient.	Believe that other oncologists are referring all eligible patients to the service; it is an expectation of practice	Tick box for service is embedded into a currently existing workflow and does not require an extra step.	Expectation that ticking the box will result in patient attendance at the Co-LEC, which will positively impact their treatment experience.
PO.4. Oncologist checks in with patients during follow-up appointments to ask about exercise progress	Describe how the Co-LEC referral process works	Believe that other oncologists are checking in with patients about exercise progress; it is an expectation of practice	Information regarding patient progress at the Co-LEC is located in an area of the patient information that the oncologist regularly accesses.	Expectation that patient check-ins will provide meaningful feedback about their experience with the Co-LEC which can result in an improved treatment experience.

*PO* Performance objective; *Co-LEC* Co-located exercise clinic

### IM task 3: implementation plan design

SAG members consulted the literature to identify theory-based methods to influence the determinants identified in task 2 [26]. A mix of individual- and organizational-level methods were chosen to strengthen the intervention by influencing multiple layers of the ecological framework simultaneously. Based on these determinants, implementation strategies were derived from the Expert Recommendations for Implementing Change (ERIC) strategy list [27] using the following criteria: contextual feasibility, ability to address identified determinants, and potential impact. A final list was compiled and presented to the group for consensus; the group agreed on the inclusion of eight strategies in the overall implementation plan design (Table 4).

**Table 4:** Implementation strategy overview

ERIC category	Contextual application	Determinant	Learning objective/Change objective
Implementation strategy			
**Use evaluative and iterative strategies**			
Audit and provide feedback	Identify key measures to describe Co-LEC success (for individual stakeholders and for organization). Create weekly reports to share with operations manager, who will use the information to modify the implementation as necessary and report key findings to individuals (e.g. general manager) and groups (e.g. oncologists) based on results.	Knowledge	Enhanced stakeholder awareness of program success and areas that need improvement to encourage program refinement.
**Develop stakeholder interrelationships**			
Identify and prepare champions	Identify and prepare an oncologist who will take the lead in promoting the Co-LEC implementation amongst the medical staff, overcoming indifference or resistance and liaising with the management/implementation teams to communicate the needs of the oncologists to ensure they are being met.	Group Norms	Recognition that the Co-LEC service is a part of normal operating procedures within GenesisCare.
Use an implementation advisor	Appoint a person with implementation experience and programming expertise to guide the project.	Knowledge	Understanding of implementation best practices across stakeholders.
**Train and educate stakeholders**			
Conduct educational meetings	Schedule sessions with oncologists during regularly scheduled meetings to provide training and updates regarding the Co-LEC. Organize sessions to teach each administrative group about the Co-LEC and their role in it.	Knowledge	Understanding of Co-LEC vision and overarching implementation plan.
Develop educational materials	Develop and format “how-to” information sheets to outline the steps of how the Co-LEC operates and the associated workflows.	Knowledge	Understanding of roles and responsibilities for the service.
**Utilize financial strategies**			
Access new funding/use other payment schemes	Utilize the Medicare chronic disease management plan to support the service. Update billing system to capture these payments.	Environmental facilitators	Facilitate financial sustainability of the service.
**Change infrastructure**			
Change record systems	Update EMR to include the Co-LEC, so appointments can be captured and all relevant participation information recorded.	Environmental facilitators	Facilitate the recognition that exercise is a standard component of treatment at GenesisCare.
**Support clinicians**			
Revise professional roles	Employ the AEP within GenesisCare; appoint lead PSO to schedule for the Co-LEC; include the Co-LEC tasks in job descriptions for all relevant roles.	Environmental facilitators	Facilitate better intra-organization communication regarding Co-LEC.

*ERIC* Expert recommendations for implementing change; *Co-LEC* Co-located exercise clinic; *EMR* Electronic medical record; *PSO* Patient services officer

### IM task 4: protocol and material production

In this task we designed, produced and pre-tested materials based on the methods and implementation strategies chosen in task 3 (Table 4). Since the “adopter” was a part of the SAG and participated in the decisions to re-implement the program, no materials were necessary for the adoption phase.

#### Implementation

Using information gathered through the RE-AIM evaluation, follow-up conversations with key members of each group of “implementers” (as identified by the GM), and consultation with an oncologist “program champion”, the SAG outlined proposed systems changes to integrate the Co-LEC into standard organizational workflows. Specifically, a Co-LEC tick box was added to the oncologists’ initial patient visit form that, when ticked, generated an alert for a PSO to call the patient to schedule an appointment at the Co-LEC. Additionally, a dedicated section was defined for exercise information to be entered into the patient’s electronic medical record (EMR). A presentation was prepared for the oncologists to describe and demonstrate the updates; it was included in the agenda of a regular monthly meeting prior to the re-launch of the Co-LEC. Finally, a “how-to” guide, with an introduction to the new Co-LEC operations, detailed workflows, and key contact details was created for each implementer group and given to the team leads for each implementer group for training and distribution. An official re-launch date was distributed.

#### Maintenance

Throughout the implementation design process, the GM expressed clear categories for success: patient participation, oncologist engagement, and financial stability. Using these as a guide, a monthly reporting template was created to provide feedback about each category. The operations manager was appointed as a lead for the service (by the GM) and given authority to make changes as necessary to support the vision.

### IM task 5: evaluation plan

In the final task, an evaluation plan was created to allow for ongoing refinement and improvement of the service and overall effectiveness of implementation. The RE-AIM framework was used to develop a comprehensive evaluation plan, with the aim to complete one-year after implementation. A mixed-methods approach was designed to ensure qualitative data could elucidate information generated through quantitative methods. As this project was still in its early stages, this was especially important to ensure barriers and facilitators were fully understood [28]. Data sources included audit and feedback reports, clinic records, surveys, and semi-structured interviews with the target implementers and maintainers (Table 5).

**Table 5:** Outcome measures for evaluation plan

Framework Category	What will be measured?	How will it be measured?	Why is it being measured?
**Reach**	Number of patients who received a call to book an appointment at the Co-LEC compared to number of patients eligible for the service	Clinic records	To demonstrate the integration of oncologist referral within the clinics
patient level			
**Effectiveness**	Patient enrollment in exercise program	Clinic records	To demonstrate the effectiveness of the implementation strategy in engaging patients in the Co-LEC
patient level	Patient attendance for initial consult		
**Adoption**	Number of oncologists per site that participate in exercise referral compared to those able to refer	Clinic records	To determine the absolute number, proportion, and representativeness of utilization of referral the program at both a site and individual provider and staff member level.
organizational level		Surveys	
	Number of exercise referrals completed per oncologist		
	PSO engagement in booking process		
**Implementation** (Fidelity)	Fidelity to proposed workflow	Surveys	To demonstrate adherence to the proposed workflow and highlight any deviations and/or intentional adaptations
	Program costs	Clinic records	
			To compare the patient experience to the protocol to understand what components of the intervention are actually being delivered by the oncologists.
organizational level	Patient experience		
**Maintenance**	Degree to which the practice has become integrated into standard practices for the organization and individual oncologists	Semi-structured interviews	To understand to how much a part of the routine the referral practice has become and highlight areas that may threaten its ability to be sustained
organizational level			
	Financial sustainability for service		
		Policy/workflow audit	
		Clinic records	

*Co-LEC* Co-located exercise clinic; *PSO* Patient service officer

## Discussion

This study provides a description of a systematic and iterative process used to develop an implementation plan to support a co-located exercise clinic in a private oncology setting. Employing the IM process to overcome challenges to utilization of an exercise oncology clinic within a private cancer center raised three important issues. First, context specific implementation planning allows for the identification of potential barriers and facilitators of program success. Second, the IM process provides a clear and attainable roadmap to guide the development of an implementation plan. Finally, partnership development and stakeholder selection to the planning committee are pivotal to the process of developing an implementation plan.

Overcoming the challenge of program implementation is a critical step towards exercise becoming integrated into standard oncology care. Despite the exponential increase of evidence demonstrating the value of exercise in oncology, the gap between research and practice severely limits its potential impact [29]. Programs that do not have an integrated, well-considered contextually appropriate plan are unlikely to achieve on their potential success [14]. This poses an important risk to the advancement of the field, as underutilization is often a precursor to poor program outcomes [16]. Low utilization dilutes outcomes critical to demonstrating the value of exercise oncology to providers, administrators, and payers [30], suggesting the program itself is not effective. Buy-in from these sectors is vital in creating the infrastructure necessary to bring exercise oncology into standard healthcare pathways [8] and this cannot be accomplished without robust data to demonstrate impact. The Co-LEC is an example of an effective program hamstrung by implementation issues severely impacting its utilization [13]; but it is not unique. Beidas et al. documented their process of implementing an exercise oncology research program into a community setting [31]. They also encountered barriers negatively impacting utilization, including lack of oncologist engagement. Despite this, few examples exist of exercise oncology programs engaging in robust implementation planning.

The IM process provided an appropriate roadmap to guide the development of an implementation plan to support the Co-LEC. While it has been established that evidence-based implementation plans are an integral component of successful translational research [14], there remains limited guidance about how to select and tailor implementation strategies appropriately [27]. The development of an implementation plan for the Co-LEC required a process that was evidence-based yet could be operationalized to keep all stakeholders engaged. The IM’s systematic approach allowed stakeholders to see the logical progression of the process from the outset. The inclusion of an implementation expert was critical, as it provided familiarity with the core principles of implementation science that served to focus the direction of the team. As calls are being made to include implementation as part of effectiveness trials in the future [32], the IM process is well suited to meet this need. Its step-by-step guidance allows a group with diverse expertise to create a contextually appropriate implementation plan, and its iterative process encourages continual improvement [15]. Furthermore, it was designed specifically to be developed in conjunction with program development [16].

Implementation research is dependent on strong partnerships to be successful. For research to be translated into clinical settings, organizations must be willing to allow systemic changes that will impact their delivery of service, and potentially key business outcomes [33]. For example, when considering methods to change physician behaviors, Grol emphasizes that individual doctors cannot be expected to change without corresponding changes in healthcare teams and the overall organization [34]. This was true for the Co-LEC, with oncologists suggesting referrals needed to be built into usual workflows to be effective. Updating workflows required organizational-level system changes and had implications for service outcomes beyond the Co-LEC. Strong partnerships must be developed to elicit this level of trust between organizations and healthcare researchers. Developing partnerships is an important component of implementation success as functional partnerships can take several years to develop [35]. The partnership between EMRI-ECU and GenesisCare had been fostered over nearly a decade. The organizations collaborated on several research studies over the years that were integral to the initial development of the Co-LEC. The belief in exercise among the oncologists and the trust in the research team by the leadership played a central role in the successful engagement of GenesisCare. This enabled an openness to re-implementing, rather than eliminating, a program that was underperforming.

Furthermore, the strong partnership facilitated participation of key senior-level stakeholders in the project. The IM protocol requires a deep understanding of the organizational structure of the adopting agency and access to key stakeholders throughout the development process [16]. Organizational change is inherent in IM. Lewin’s three-stage model of change theory describes this step as “unfreezing” whereby organizations need to both determine what needs to change and create a need for change to happen; communication between program end-users and program planners is critical during this phase [36]. Recognizing this when choosing SAG members is important to facilitate the “unfreezing” process. For the Co-LEC implementation planning, members of the SAG included a senior oncologist and the regional head of marketing at GenesisCare. They served as a linkage system to ensure the needs of the program end users were appropriately considered during the planning process [37]. Moreover, their demonstrated ability to be influential within the organization allowed for generation of support for the project in anticipation of the implementation [25]. Additionally, the regional GM of GenesisCare served on the SAG. As buy-in and support of senior management is nearly always required for implementation projects to be successful [25], and his involvement was critical. Because he was an active participant in the process, he was engaged in the program and had direct authority to enact changes immediately, bypassing the proverbial “red tape” often involved in organizational change. This saved the committee time and resources and set the project up for success as it moved to the next “change” phase of organizational change, where leadership engagement and motivation are critical components of success [36].

### Strengths and limitations

This is the first paper to describe the in-depth process of developing an implementation plan for an exercise clinic within a private oncology center. Strengths of this work include participation from a well-established healthcare organization with prior experience managing an exercise program. This brought real-world issues inherent in translational exercise oncology to light and allowed for immediate application of the work. Additionally, all domains of the ecological framework were considered during the process and strategies at both the individual and organizational levels were recommended. Given the profile of the key stakeholders, the opportunities to engage the entire group were limited. Lastly, the need to balance the real world demands of an operational organization with project needs required the team to adhere to a tight timeline and limited the time to engage in each task.

## Conclusion

The IM protocol provided a roadmap to guide development of a comprehensive implementation plan that considered all ecological domains, was informed by theory, and demonstrated an extensive understanding of the implementation context. Strong research-practitioner partnerships and effective stakeholder engagement were critical to development of the plan. As the field of exercise oncology moves toward routine clinical integration, the IM process can enhance program impact as it provides a clear, step-by-step method to ensure optimal programming is incorporated as part of overall program planning. Future work should investigate the feasibility of incorporating IM as an integral component of program planning and including implementation content in degree curricula and professional development courses for exercise professionals.

## Data Availability

Data sharing not applicable to this article as no datasets were generated or analysed during the current study.
